# Not Your Mother’s Melanoma: Causes and Effects of Early Melanoma Diagnosis

**DOI:** 10.3390/dermatopathology9040043

**Published:** 2022-11-27

**Authors:** Kaviyon Sadrolashrafi, David Graham Cotter

**Affiliations:** 1 Department of Internal Medicine, Kirk Kerkorian School of Medicine at UNLV, Las Vegas, NV 89106, USA; 2 Las Vegas Dermatology, Las Vegas, NV 89144, USA

**Keywords:** melanoma, skin cancer, melanoma incidence, melanoma staging, precision medicine, GEP, NGS, FISH, CGH

## Abstract

The year 2022 will herald approximately 100,000 new cases of cutaneous melanoma (CM), and over 7000 deaths from CM. Over the past 40 years, CM incidence has increased nearly six-fold; however, annual mortality has remained relatively constant. These trends encapsulate the phenomenon of overdiagnosis. Increased recognition of indolent lesions that appear histologically malignant may be leading to a melanoma epidemic. Enhanced melanoma awareness, screening efforts, physician uncertainty, medical-legal pressures, and diagnostic scrutiny using tools like immunohistochemical staining, mole mapping, dermoscopy, confocal microscopy, and molecular diagnostics contribute to increased CM diagnosis. As a result, current melanoma staging and treatment guidelines are being challenged. Existing standards fail to accurately identify histologically benign lesions that are lethal or, conversely, histologically malignant lesions that are innocuous. Healthcare systems and, more importantly, patients suffer from this diagnostic ambiguity that leads to the over-treatment of innocuous melanomas and under-treatment of aggressive melanomas. As dermatology continues to experience a shift towards earlier diagnosis of melanoma, management strategies must adapt. Herein, we review factors that may contribute to the increased incidence of melanoma, emphasize deficiencies in current staging systems, and provide insights into the future of melanoma management via precision medicine.

## 1. Introduction

Cutaneous melanoma (CM) is a potentially fatal malignancy [1]. United States population-based projections estimate that nearly 100,000 new cases will be diagnosed in 2022, leading to over 7000 deaths [2]. CM incidence has risen approximately six-fold over the past 40 years, while annual mortality remains stable [3]. The increased incidence of CM coupled with stable mortality embodies the epidemiological concept of overdiagnosis—the identification of histologically malignant yet biologically indolent lesions [4,5]. Over time, this trend towards cancer overdiagnosis has been observed across various cancers, including breast, renal, prostate, and thyroid cancer [6,7]. Enhanced rates of early-stage melanoma diagnoses, which we define as melanoma in situ and stage T1a tumors (<0.8 mm thick and without ulceration), may result from increased melanoma awareness, screening efforts, and expanded diagnostic scrutiny via mole mapping, dermoscopy, confocal microscopy, immunohistochemical staining, and molecular diagnostics, creating proportions that some term an “epidemic” [8]. The increased incidence may also reflect provider hesitancy in missing cases due to medical-legal pressures, leading to an overly inclusive diagnosis with defensive undertones [4,9]. As a result, paradigms as banal as skin cancer screening and as carefully curated as melanoma staging and treatment guidelines are being challenged. As dermatology continues to experience a shift towards earlier diagnosis of melanoma, management strategies must adapt. Herein, we review factors that may contribute to the increased incidence of melanoma, highlight deficiencies in current staging systems, and offer insights into the future of melanoma management via precision medicine.

## 2. Putative Reasons for Increased Incidence of Melanoma

The National Cancer Institute’s Surveillance, Epidemiology, and End Results (SEER) Program was designed in 1973 to provide nationwide statistical information about annual cancer diagnoses [10]. Since its establishment, CM incidence rates have risen tremendously. A key distinction often lost in the staggering metrics its registries produce is whether increased incidence accurately depicts rising disease burden or if efforts to screen for and “not miss” the disease have led to inflated case numbers [11]. While the SEER Program expertly captures data, such a population-based registry cannot identify underlying causes that drive the disease burden. Therefore, further exploration of factors contributing to enhanced CM incidence, as broadly delineated by national cancer registries, is warranted.

Myriad genetic and environmental factors contribute to CM development. As significant genetic drift towards a stronger melanoma predilection within the United States population over the last 40 years is unlikely, behavioral, sociologic, and iatrogenic etiologies likely underpin increased CM incidence. For example, overexposure to intermittent ultraviolet (UV) radiation partially contributes to increased CM incidence [12]. Sun-seeking behaviors like sunbathing and indoor tanning have increased over the last 40 years, but not enough to entirely account for the increased incidence of CM alone [13,14]. Welch et al. (2021) contend that the association between CM and exposure to UV radiation cannot account for the observed increase in incidence over these 40 years, as the relationship carries a relative risk of 2. Under these parameters, CM incidence would be expected to—at worst—double during four decades, yet incidence has sextupled [4]. Age-adjusted incidence rates support their postulation. Using the SEER registry, Lashway et al. (2021) found that CM incidence increased with age between 1975 and 2017. After adjusting for age and stratifying groups into 5-year birth cohorts, these researchers discovered significantly greater melanoma incidence at older ages [15]. Paulson et al. (2019) further this age-based dichotomy when reporting that CM incidence decreased in populations under 40 years old between 2006 and 2015 but increased in people 40 years old and over during the same time [16]. The discrepancy between age groups and CM incidence may reflect biases in diagnosing CM. For example, some providers are unwilling to diagnose melanomas in children, despite meeting histological parameters compatible with CM in elderly populations. Others are more averse to performing biopsies on children due to their low index of clinical suspicion and thus wait for clinical evolution to prompt procedural intervention [8]. Undoubtedly, an aging population partially contributes to increased CM incidence as well. Recent estimates suggest that population growth and increased life expectancy will catalyze new CM diagnoses since melanoma risk, like other cancers, grows with age [17].

## 3. Increased Skin Cancer Awareness

Overarching shifts in diagnostic approaches and attitudes contribute to increasing CM incidence. The steep increase in biopsy and diagnosis rates of thinner melanomas, in particular, has augmented the total number of tumor cases identified [4,9,18,19,20]. Critical contributors to these observed trends include increased skin cancer awareness by patients and providers and efforts targeting primary and secondary prevention at the local and national levels [15,18,20]. Before professional associations like the American Academy of Dermatology endorsed melanoma screenings, CM diagnosis generally revolved around the presence of distinguishable macroscopic findings like size and ulceration; as a result, the disease was often identified during its later stages [15].

The establishment and propagation of the ABCD (eventually ABCDE) criteria for the gross inspection of cutaneous lesions in the mid-1980s emphasized the importance of self-screening via skin checks and empowered individuals to become active partners in their care [21]. The development of national public education campaigns has encouraged sun-protective behaviors and taught individuals—particularly school-aged children—about the significance of early CM detection [22]. Adoption and expansion of these public health initiatives, broadening media exposure, and advertisements of preventative modalities by pharmaceutical companies have heightened melanoma awareness within populations in the United States and other countries [8,15,22]. Australia, for example, implemented heavily-publicized, statewide campaigns emphasizing the importance of sun safety and early melanoma detection in the early 1980s. These coordinated, preventative efforts increased public understanding of CM. They triggered a public desire for skin cancer screenings and more accessible clinics designed explicitly for skin cancer detection throughout the country [22]. Similarly, the American Academy of Dermatology’s SPOTme skin cancer screening program has identified thousands of melanomas since its inception in 1985, many of which would likely have gone undetected [23]. This public campaign targets barriers to healthcare access, namely poor health literacy and lack of insurance, providing over 850,000 individuals who are otherwise unlikely to see a dermatologist with free skin cancer screenings from 1986 to 2014 [23]. Prevention initiatives are essential to mitigating melanoma morbidity and mortality, lessening the burden on individuals and healthcare systems [24].

Changes in public awareness of melanoma have likely increased the number of CM cases diagnosed. However, managing these lesions creates unintended consequences that can hinder the beneficial aspect of early detection. Biopsy of potentially indolent thin melanomas unlikely to metastasize and lead to death generates a vicious cycle that worsens the quality of healthcare delivery and a patient’s quality of life. In addition to the physical (e.g., scars and infections), financial (e.g., out-of-pocket costs from frequent surveillance), and psychological (e.g., anxiety and depression) toll that over-treatment causes, unnecessary procedures may interfere with healthcare access for symptomatic patients who need care the most [4,20,22]. Increased incidence of early stage disease with stable mortality rates suggests that treatment has negligibly impacted an individual’s chance of survival in many cases [22]. These data trends imply that the actual occurrence of melanoma has remained relatively constant over time; greater incidence of late-stage disease and higher mortality rates would be expected if CM incidence was genuinely increasing. Instead, an increase in early stage disease is being observed without the corresponding increase in deaths [4]. Greater early stage CM incidence may reflect more aggressive and extensive screening initiatives [9]. Perhaps indolent melanomas are ubiquitous and remain undiagnosed until they present more aggressively or are evaluated through a more critical diagnostic lens.

Either way, implemented diagnostic standards only take us so far. Pigmented lesions may episodically resemble CM, and some tumors spontaneously regress altogether [5,8]. It is difficult to predict which lesions will progress and which will not. Current clinical diagnostic processes generally evaluate melanocytic lesions based on their morphological qualities using the ABCDE criteria [21]. Despite using other adjunct modalities to enhance clinical judgment, diagnostic certainty is challenged when dermatologists encounter non-prototypical lesions. Many diagnoses ultimately suffer from this ambiguity, which elucidates physician variability in clinical practice and clinical recognition, ultimately leading to more biopsies [4]. Diagnostic ambivalence can be extrapolated to dermatopathologists, as unconventional and complicated melanocytic lesions tend to be “overcalled” as CM instead of “undercalled” as benign lesions [8,9]. Alas, a significant issue plaguing current diagnostic guidelines and treatments is the relative inability to differentiate benign, atypical melanocytic lesions from malignant lesions at risk for metastasis/progression [22]. This understanding has caused over-treatment of early stage, innocuous melanomas that are unlikely to lead to death and under-treatment of early stage, biologically aggressive melanomas that contribute to overall melanoma-associated mortality. The five-year melanoma-specific survival (MSS) rate of patients with stage I melanoma is 98%; therefore, we consider the 2% of stage I melanomas that lead to death as these early aggressive melanomas [25]. Pathological stage tremendously impacts a patient’s likelihood of survival, as five-year MSS rates decline from 90% to 77% when one increases pathological from stage II to stage III melanoma [25]. However, striking nuances within these trends are evident upon closer inspection when measured over ten years. For example, the ten-year MSS of stage IIIA and IIIB melanoma is 88% and 77%, respectively, which sharply contrasts with the 82% and 75% MSS of stage IIB and IIC melanoma [25]. The relative aggressiveness of CM also varies based on its histological subtype, with nodular melanoma contributing more to CM-related deaths than other subtypes like acral lentiginous and lentigo maligna [26]. Accurate recognition of metastatic potential can decrease mortality via the early intervention of aggressive melanomas while simultaneously reducing societal and individual costs associated with the potentially excessive treatment of more indolent lesions [27].

## 4. Pathologic Diagnostic Tools

A significant conundrum affecting CM diagnosis is the lack of a conclusive histopathological gold standard, particularly for ambiguous thin lesions [9]. Immunohistochemistry (IHC) has served as a tool to help differentiate CM from benign lesions and other tumors through the identification of characteristic markers expressed by melanocytic lesions. Lezcano and colleagues (2018) performed an IHC analysis for preferentially expressed antigen in melanoma (PRAME) protein expression in melanocytic tumors and found that 83.2% of primary cutaneous melanomas regularly and diffusely expressed PRAME [28]. Their findings align with the research of Ikeda et al. (1997), which found that 91% of melanomas in their mRNA studies expressed the PRAME gene [29]. Although diffuse PRAME immunoreactivity in melanocytic lesions is highly suggestive of CM, PRAME expression alone is insufficient for a conclusive melanoma diagnosis. Lezcano et al. (2018) implore that IHC for PRAME is used as a supplementary diagnostic tool [28]. Mihic-Probst et al. (2005) studied the expression of p16, a protein characteristically found in the nucleus that negatively regulates the G1/S checkpoint of the cell cycle, to help distinguish benign nevi from CM. IHC staining for this protein proved helpful, as its localization outside the nucleus enhances the likelihood of a potential CM diagnosis. The absence of IHC staining for p16 is another vital phenomenon, as 30% of melanomas lost p16 staining entirely [30]. Unfortunately, these immunohistochemical markers are not definitively associated with enhanced prognostic value, thus limiting their utility to clinical practice [31]. Barring metastasis, the absence of a true biological paradigm predicting the disease burden of challenging and atypical melanocytic lesions generates uncertainty. It underscores an inherent quality of many “final” diagnoses made today—their ultimately subjective interpretation [8]. The subjectivity in histopathological diagnosis reflects discordance between experts, which further emphasizes the biologic variability of specific lesions and the difficulty in reaching an accurate CM diagnosis using current standards [32].

Farmer and colleagues (1996) depict the challenges encountered during a histopathological evaluation when attempting to differentiate CM and melanocytic nevi; an assessment of 37 specimens by expert pathologists resulted in one discordant diagnosis in 62% of samples and at least two conflicting interpretations in 38% of samples [32]. In a more contemporary, large-scale analysis involving 187 United States-based pathologists, Elmore et al. (2017) test CM diagnosis reproducibility and accuracy using 240 skin biopsies. Specimens were stratified into five classes based on their histological classification as per the Melanocytic Pathology Assessment Tool and Hierarchy for Diagnosis (MPATH-Dx). Groups ranged from melanocytic lesion/mild atypia (Class I) to late-stage, invasive melanoma (Class V). Lesions placed into higher classes reflect a greater perceived risk for progression. These researchers evaluated discordance between the same provider at different times (intraobserver analysis), providers against their colleagues’ diagnoses (interobserver analysis), and providers against a consensus reference diagnosis made by an expert panel of dermatopathologists. Their study found more significant intraobserver discordance (65%, 40%, and 37%) and interobserver discordance (75%, 55%, and 54%) for ambiguous melanocytic lesions encompassing moderately dysplastic nevi (Class II), melanoma in situ or severe atypia (Class III), and early stage invasive melanoma (Class IV), respectively. This study also found discordance rates greater than 40% after comparing diagnoses of lesions in Classes II-IV to the consensus reference standard [33]. Piepkorn et al. (2022) support these observations in their study investigating intraobserver reproducibility; these researchers found more significant diagnostic discordance (and thus less reproducibility) when moderately dysplastic nevi and melanoma in situs were analyzed than when mildly dysplastic nevi and invasive melanomas (at least stage T1b) were analyzed [34]. These findings further the notion that melanocytic lesions on extreme ends of the diagnostic spectrum (i.e., clearly benign lesions or CM) are relatively undisputed histologically and often generate less diagnostic discordance. This idea is essential, considering that some of the most frequently biopsied lesions include melanocytic lesions that fall into this range like, dysplastic nevi, melanoma in situ, and invasive melanomas [34]. Collectively, these data illustrate a troubling reality: as melanocytic lesions become more indeterminate and unconventional, diagnostic consistency and precision amongst reviewing physicians diminishes.

The uncertainties and inconsistencies associated with CM diagnosis—even when made by practitioners with the highest levels of training—have caused many physicians to reduce their pathological diagnostic threshold [4]. Frangos et al. (2012) encapsulated this diagnostic drift by reviewing skin-biopsy samples diagnosed two decades before their study. These researchers observed trends that align with lower pathological diagnostic thresholds, discovering that dermatopathologists generally reclassified diagnoses previously deemed benign as malignant, but rarely downgraded a CM diagnosis to benign [35]. In some instances, the dermatopathologist reviewing the same slide they interpreted twenty years ago changed their initial diagnosis from benign to CM [4,35]. These findings are even supported in the short term, as re-examination can alter the initial diagnosis of an identical slide being reviewed by the same physician as little as eight months later [33]. These behaviors reflect the evolution of diagnostic standards over time, which Cazzato et al. (2021) capture in their description of the repeated reclassification of animal-type melanoma (ATM) [36]. Initially described in humans in 1979 by Levene, ATM is distinguished by the periappendageal growth of spindle, epithelioid, and dendritic cells containing large amounts of melanin [37]. Lenient morphological criteria available at the time of its inital classification led to the ATM designation. As the biological behavior of the lesion was better understood, and general histopathological nomenclature became more specific, the clinicopathological entity was renamed numerous times to provide a more accurate description, most recently in the WHO Classification of Skin Tumors as pigmented epithelioid melanocytoma (PEM) [36]. This example typifies the plausible influence of changing histopathological diagnostic criteria on increased melanoma incidence.

Another factor likely contributing to lower CM diagnostic thresholds is medical-legal pressures that put a significant onus on physicians not to miss a melanoma [9]. Due to fears of medical malpractice litigation, many physicians practice defensive medicine during their evaluation of melanocytic skin lesions [38]. Titus et al. (2018) found that nearly one in four pathologists diagnose melanocytic skin lesions more severely due to litigation concerns over a missed diagnosis [38]. Provider hesitancy in missing cases lowers thresholds to refer, biopsy, and diagnose. This fear-based, vicious cycle of overdiagnosis often comes at the expense of patients; these defensive behaviors reduce exposure to malpractice litigation, but patients do not always benefit clinically [39]. Rayess et al. (2017) found that lawsuits over an alleged melanoma misdiagnosis were the most common cause of legal action against dermatologists and pathologists. Most malpractice cases were resolved through payment, and the presence of misdiagnosis resulted in an average payout greater than $750,000 [40]. The prosecution of dermatologists generally revolved around the improper evaluation of pigmented lesions, while the prosecution of pathologists centered on the failure to diagnose CM through histopathological analysis [40]. Malpractice concerns—not medical ones—lead some providers to order unneeded assessments even though they are aware of and acknowledge the possible harm that additional testing may cause patients [41]. 

## 5. Clinical Diagnostic Tools

Various diagnostic modalities have been developed to facilitate early CM detection in the clinical setting. Tools like dermoscopy, mole mapping, confocal microscopy, and pigmented lesion assays enhance the ability to diagnose CM and are especially useful when encountering ambiguous melanocytic lesions [8]. Dermoscopy (epiluminescence microscopy) relies on magnifying skin lesions with devices that minimize light penetration to allow for in-depth inspection of microscopic structures [42]. Enhanced visualization allows this non-invasive technique to distinguish benign and malignant lesions better than the naked eye, as many lesions lack prototypical features; furthermore, increased diagnostic certainty mitigates the need for excisional biopsy in many cases [43,44]. A meta-analysis found that this technique had a relative diagnostic odds ratio of 9 compared to a naked eye examination, suggesting that the odds of accurately diagnosing CM are much stronger when utilizing dermoscopy in the clinical setting [43]. The strength of this tool is supported by Carli et al.’s (2004) findings of dermoscopy enhancing the quality and quantity of biopsies through an improved malignant-to-benign ratio of excised lesions [44]. Mole mapping of the entire skin surface, often used as an adjunct to traditional dermoscopy, documents suspicious lesions through a series of high-quality photographs [45]. This non-invasive modality makes melanoma surveillance more precise and objective, as it establishes a baseline for pigmented lesions and dynamically assesses their progression over time. Continuous evaluation of lesions captures a central feature of malignant melanomas—their evolution. A baseline image for the whole body is also created, which can be used for comparison to help identify novel lesions [45]. Confocal scanning laser microscopy (CSLM) exploits the inherent reflective property of subcutaneous structures like melanin granules and melanosomes to create high-resolution optical subsections that visualize the histological architecture of the epidermis and upper dermis [46,47]. Clinical benefits of this technique include its high positive predictive value, as the visualization of different layers of the skin occurs at resolutions comparable to standard pathological evaluation [47]. Pigmented lesion assay (PLA) utilizes an adhesive patch to harvest thin stratum corneum samples non-invasively and risk stratify tissue specimens based on their expression of two genes associated with CM. Collecting and focusing on biological activity helps augment clinical decision-making through its high negative predictive value, potentially lessening the unnecessary need to biopsy benign lesions [48,49].

Heightened diagnostic scrutiny using these various clinical techniques certainly contributes to increased CM incidence, so it’s important to consider whether or not they are accurate in their diagnoses. Dermoscopy can provide a robust clinicopathologic correlation for a CM diagnosis; however, its utility is invariably tied to the expertise of its user, with diagnostic sensitivity decreasing in inexperienced physicians [42]. Unfortunately, experience alone cannot guarantee an accurate diagnosis either, as early melanomas are still missed by physicians accustomed to dermoscopy [48]. The reliability of mole mapping is impacted by the pigmentation of photographed lesions, as hypopigmented melanomas are more likely to go undetected than darker lesions [50]. CSLM suffers similar limitations as dermoscopy, as its efficacy is dependent on the experience of the physicians using it. Furthermore, CSLM devices can be costly and infeasible to use widely in clinical settings [47]. PLA does not suffer from similar barriers as the previous three modalities since it does not rely on visual characteristics to diagnose CM. However, the two-gene assay does not function in specific anatomical locations, which limits its diagnostic power [48]. Additionally, since the assay relies on gene expression, its negative predictive value is lessened by mutations in the profiles of interest.

## 6. Precision Medicine

A critical challenge emerges as CM incidence rises and physicians shoulder a greater onus to detect early-stage melanomas: to accurately distinguish CM from atypical melanocytic lesions that do not pose a metastatic risk. Current TNM staging systems developed by the American Joint Committee on Cancer (AJCC) rely on tumor thickness, presence of ulcerations, and sentinel lymph node positivity to predict the metastatic potential of melanocytic lesions [51]. Despite using these criteria, early-stage (i.e., stages I and II) CM patients unlikely to develop advanced disease with early intervention still do, and patients with intermediate-stage (i.e., stage III) CM have variable prognoses [51]. Therefore, staging criteria must be augmented to reflect the metastatic potential of innocuous-appearing, thin melanomas or presumed aggressive thick melanomas that follow a more indolent course. Physicians require new staging tools to refine the array of metastatic potential exhibited by lesions diagnosed as melanoma.

Non-morphologic, biological characteristics are impartial findings and, therefore, less susceptible to the subjective pitfalls of visual diagnostic measures. To strengthen clinical acumen, the future of melanoma management must integrate precision medicine into existing diagnostic criteria to optimize the quality of healthcare delivery to patients who face a life-altering diagnosis (or misdiagnosis). Gene expression profiling (GEP) is a potential solution that evaluates melanocytic lesions at the molecular level. This prognostic test utilizes reverse transcription polymerase chain reaction to identify and amplify differentially expressed genes in high-risk (for metastasis) and low-risk tumors, thus establishing metastatic risk profiles [51,52]. GEP has been shown to identify patients with a greater metastatic risk than suggested by AJCC staging alone [52]. Next-generation sequencing (NGS) is a genomic technology developed to recognize known melanoma mutational sequences via circulating tumor DNA harvested from plasma instead of DNA obtained via melanocytic lesion excision. NGS minimizes the need for invasive biopsies and enables identification of gene sequence variation between wild-type and disease states [53,54]. Using genomic sequences specific to CM lesions, NGS can potentially predict the biological behavior of a tumor and monitor its response to treatment in real-time [53,55]. NGS proves especially useful in diagnosing challenging CM lesions that have lost characteristic melanocytic IHC markers, classified by Cazzato et al. (2021) and others as “dedifferentiated melanoma” [56]. The identification and expression of the transcription factor MITF, in particular, signals the potential for aberrant biological activity by tumor cells and consequentially poor response to targeted therapies against cancer cells [56]. Genetic analysis with fluorescence in situ hybridization (FISH) can aid in the differentiation between a benign melanocytic lesion and CM.

FISH uses colored probes targeting recurrent chromosomal abnormalities (i.e., specific DNA sequences) associated strongly with CM to discern most melanomas from benign lesions [57]. A significant drawback of this technique is that its sensitivity is predicated on the melanoma subtype in question. The subtypes differentially express affinity for the probes being used since certain chromosomal irregularities (and thus the probe’s subsequent binding strength) may not be equally represented between the different CM subtypes [58]. Nodular and acral lentiginous melanomas appear to be subtypes with the highest sensitivity because of their strong association with chromosomal irregularities. Superficial spreading melanoma and lentigo maligna show less sensitivity when being evaluated by FISH [58]. Comparative genomic hybridization (CGH) also detects chromosomal abnormalities by searching for variations in DNA copies (i.e., amplifications or deletions on chromosomes) between the tissue specimen and human control samples [59].

## 7. Conclusions

In closing, the melanoma epidemic is a genuine phenomenon heavily influenced by current pressures to not miss melanoma and to diagnose melanoma as early as possible—two goals of extreme merit that inadvertently result in overtreatment of many lesions due to the limitations of current staging guidelines for CM. Numerous factors contribute to this inescapable reality, such as expanded melanoma awareness, intensified screening efforts, greater provider hesitancy, and enhanced diagnostic scrutiny with lower diagnostic thresholds. More lesions—namely early-stage, thin lesions—are being diagnosed as CM, yet annual mortality remains relatively stable. This seemingly discordant observation raises the potential of disease overdiagnosis. A deeper exploration of CM incidence rates reveals an inconvenient truth: current staging systems rely almost exclusively on morphological characteristics and cannot accurately predict the metastatic potential of atypical melanocytic lesions or account for the influx of early melanomas. Our diagnostic standards cannot substantiate an explanation for histologically benign lesions that are lethal or, conversely, histologically malignant lesions that are innocuous. Physician are thus forced into an equivocal and unsure predicament, causing many to err on the side of caution when diagnosing CM. Diagnostic uncertainty underlies the perception of indeterminate lesions as malignant and not benign so that a fatal melanoma is not missed. This tendency paradoxically over-treats innocuous melanomas and may also under-treat aggressive melanomas, causing patients and healthcare systems to suffer. We postulate that many early-stage melanomas progress slowly, pose a minimal metastatic risk, and will remain early-stage melanomas for years, even if they are not preemptively removed through surgery. Staging criteria must adapt to account for an increased number of earlier melanomas because to address this significant dilemma, physicians need better tools to elucidate the metastatic risk of early melanomas.

The utility of precision medicine in managing melanoma extends beyond its diagnostic ability. Instead, molecular tools like GEP, NGS, FISH, and CGH should serve as adjuncts that supplement traditional melanoma staging guidelines. When used in tandem, the classification of CM becomes more comprehensive. In the case of melanocytic lesions with potentially conflicting (or absent) morphologic features, these techniques can enhance the diagnostic and prognostic judgment of even the most experienced physicians. A complete clinicopathologic picture of melanocytic lesions, especially early-stage lesions, allows physicians to accurately diagnose CM with greater confidence instead of over diagnosing out of fear of missing the malignancy. These shifts in diagnostic paradigms will lessen the psychological, physical, and financial strain placed on patients and healthcare systems by incorrect diagnoses and unnecessary invasive procedures. Most importantly, patient care will improve when the right diagnosis leads to the right treatment at the right time.

## Data Availability

The data presented in this study are openly available in MEDLINE at doi:10.1016/S0046-8177(96)90157-4 [32], doi:10.1136/bmj.j2813 [33], and doi:10.1111/j.1365-2133.2008.08713.x [43].
